# Self-Regulated Learning Practice of Undergraduate Students in Health Professions Programs

**DOI:** 10.3389/fmed.2022.803069

**Published:** 2022-02-16

**Authors:** Ebenezer Chitra, Norul Hidayah, Madawa Chandratilake, Vishna Devi Nadarajah

**Affiliations:** ^1^School of Health Sciences, International Medical University, Kuala Lumpur, Malaysia; ^2^International Medical University (IMU) Center for Education, International Medical University, Kuala Lumpur, Malaysia; ^3^Faculty of Medicine, University of Kelaniya, Ragama, Sri Lanka; ^4^IMU Center for Education and School of Medicine, International Medical University, Kuala Lumpur, Malaysia

**Keywords:** self-regulated learning, undergraduate, health science, academic goals, personal development

## Abstract

**Background:**

University students are expected to take charge of their learning without being dependent on teachers. Self-regulated learning (SRL) is the process by which students direct their learning to achieve their set targets and goals in a timely and controlled manner. This study was undertaken to explore the practice of SRL by undergraduate students from different programs in a health science focused university during COVID-19 pandemic.

**Method:**

Thirty-three undergraduate students of five health professions education programs were recruited to take part in focus group discussions to explore their SRL practice with online learning. Their responses were subjected to thematic analysis.

**Result:**

Our students appeared to practice SRL, going through the phases of forethought and goal setting, performance and self-reflection. They set goals for academic as well as personal development in the university. Academic goals like achieving target GPA or marks were achieved by following different study techniques, personal management including time management, and by creating a conducive learning environment. Personal development such as interpersonal skills, social networking was achieved through socializing and participating in extracurricular activities. The students also engaged in self-reflection and analysis of their own performance followed by designing strategies to manage the challenges they faced.

**Conclusion:**

Undergraduates of health professions programs appear to show evidence of practicing SRL. Although impacted by COVID-19 induced lockdown and online learning, they seem to have strategized and achieved their goals through individualized SRL processes. Promoting and fostering an atmosphere of SRL in universities to cater to the needs of the students would help them be more successful in their careers.

## Introduction

Self-regulated learning (SRL) is the cognitive process through which a learner regulates his/her own learning by analyzing the task, setting clear learning goals, planning out the processes, enacting the process with self-control, reflecting on own performance and making the necessary improvements to achieve the set goals (1). Self-directed learning (SDL), on the other hand, is the process by which the student initiates and directs his/her own learning (2). SDL is more related to adult education whereas SRL is a broader concept that is applied more in school and college education scenario (3). Although self-regulated learning is deemed to be a determining factor for academic achievement (4), the percentage of students from universities as well as schools who practice high level of SRL is generally low (15–25%) across different countries (5). Students immensely benefit by adopting SRL having learnt self-efficacy, motivation and empowerment through SRL (6), and it promotes lifelong learning (7).

However, SRL is limited by the challenges of adopting it. Personal, contextual and social attributes are shown to affect the practice of SRL by the students (8, 9). Personal attributes include emotion control, psychological status, motivation, metacognition, attention, and effort control. Negative emotions and stress are shown to deter students from practicing SRL (10). Contextual attributes are curriculum, facilities, atmosphere, environment, instructors etc. Social attributes relate to people, peer, family background, the experience, and the relationship. Some challenges that are reported to prevent students from adopting SRL are time limitation, reluctance to adopt new methods of study, unwillingness to change their outlook, a restrictive assessment-driven curriculum, etc. (5). Academic diversity in terms of academic orientation and commitment varies widely among the students resulting in a gap between students who are motivated toward active learning and those who do not have clear academic goals (11).

Provision of the right learning environment and support can promote the development of SRL in students of different disciplines. Novice medical students in clinical settings require active help from peers and supervisors to facilitate their learning (12) and their practice of SRL transition from pre-clinical to clinical setting (13). SRL is associated with success in clinical skills of medical students (14). Clinical reasoning skills can be developed in nursing students through methods to promote self-regulation through self-observation and self-monitoring (15). Teaching approaches can also help development of self-regulation by the students thereby leading to improvement in general academic performance (16). This would eventually lead to life-long learning and help students' professional advancement.

COVID-19 pandemic and the resulting shift to online learning majorly impacted student learning practices globally. This led to educational disadvantage for some students more than the others depending on their level of self-regulation and motivation. The students had to make fundamental changes to their learning style to fit in the unchartered territory of remote learning where they experienced limited support by the faculty or peers (17). This led to increased reliance on their individual SRL practices. Distance learning requires more self-regulation than regular learning since it minimizes supervision and support from faculty. Successful learning depended on the students' ability to manage time, tasks and cope with the lack of contact with their faculty and peers (18).

While several studies on SRL have focused on students in a specific clinical setting or health professional courses (19–21), not many studies have been done across several health professions programs offered in the same learning environment or settings. The objectives of this study are to explore the practice of SRL by undergraduate students of different health professions programs and determine its impact on academic and personal development. This study was conducted during COVID-19 pandemic and therefore gives and additional perspective on SRL practice by university students during the pandemic. The findings of this study will provide an overview of students SRL practices across programs during online learning and help guide university teachers and managers, to enhance both the academic and social environment for the students.

## Methods

### Study Design

A basic qualitative research approach was adopted in the study (22).

### Study Setting

The study was conducted in a university in Malaysia, where undergraduate programs in health professions education are offered (Medicine, Pharmacy, Nutrition Dietetics, Biomedical Science, Medical Biotechnology).

### Study Population, Sampling and Recruitment

The total populations of the student cohorts which were considered for the study were 60, 15, 60, 65, and 177, respectively. Undergraduates with 6 months of academic experience in their respective programs were considered in the study by excluding foundation and postgraduate students who were in the same cohorts of students. The participants were sampled using maximum diversity sampling technique (23, 24). This helped the sample to be a heterogeneous group of participants with diversity in terms of socio-demographic characteristics. The inclusion criteria of (a) Undergraduate students, (b) Diverse background in terms of gender, pre-university qualification, local/international students and exclusion criteria of (a) Foundation or post-graduate students, (b) Students from first semester fresh into university. A briefing was given to the student cohort and volunteers contacted the researchers for participation.

### Data Collection

The data were collected using Focus Group Discussions (FGD). FGD allowed in-depth exploration of the research question and for a collaborative discussion (25). Five FGDs participated by 5–8 students each were conducted online using Microsoft Teams. All the students who volunteered were invited and all participated in the FGDs. FGDs were guided by an interview protocol with semi-structured questions, which facilitated in depth exploration of self-regulated learning practices (26). The questions were “*what were your aims or goals when you came to the university?*,” “*what was your strategy for achieving it?*,” “*what is your study style?*,” “*what are the factors that affect your learning?,”* “*how has the pandemic and online learning affected your studies?*” and “*how do you respond to success or failure in reaching your goals?*” The average duration of the FGD was 60–90 min. All FGDs were conducted in English, and they were audio recorded.

### Data Analysis

The focus group discussions were transcribed *ad verbatim* and subjected to thematic analysis using the Braun and Clarke model for thematic analysis using inductive approach; the FGD transcripts were thoroughly read multiple times for familiarizing with the data, codes were identified, themes were formed, reviewed, defined and compiled into a report (27). The data were open-coded and initial codes were generated manually by the researchers under different categories. During second coding, the codes from all the five FGDs were assembled and compared. Conceptually similar codes were grouped together. During third coding, the codes were cleaned for identical codes implying the same meaning. The categories were reviewed, common concepts or processes were identified, and three themes and seven sub-themes were generated. The data for different themes were color coded and analyzed.

### Trustworthiness

Trustworthiness of the data was established through dependability and confirmability where an audit trail detailing the process from obtaining ethical approval to preparation of dissertation was carried out (22). The results of this study might be applicable and transferrable to any other university context in Malaysia.

### Research Team and Reflexivity

The research team consisted of education researchers from an international medical institution who were varied in terms of disciplinary perspectives. The first research team member is an immunologist and researcher (EC). The second member is trained in educational psychology and teaches in Health Professions Education Program (NH). The third team member is a medical educationist with medical background (MC). The fourth team member is a biochemist and medical educationist by training (VDN). Throughout the study, the team was actively involved in the process from planning, obtaining approval, collecting, and analyzing data and writing the manuscript, while at the same time aware of own subjectivity based on different backgrounds. Henceforth, the team continuously recognized and reflected during the process to leverage on different perspectives.

Ethics approval for the study was obtained from the institutional ethics review committee.

## Findings

A total of 33 students from five different undergraduate health professions programs participated in five focus group discussions. Socio-demographic data (Table 1) revealed that the participants were of both the genders, included both local as well as international students from different pre-university programs. The findings were common for the participants from varied background.

**Table 1:** Demographics of the participants.

	**Gender**		**Country**		**Pre-University program**						
	**Male**	**Female**	**Local**	**International**	**A-levels**	Ausmat^*^	FIS^*^	UEC^*^	STPM^*^	**Matriculation**	**Others**
FGD 1 (biomedical science)	1	6	6	1	1	0	5	0	0	0	1
FGD 2 (medical biotechnology)	4	2	6	0	2	1	1	1	0	1	0
FGD 3 (nutrition & dietetics)	0	7	7	0	1	1	3	2	0	0	0
FGD 4 (pharmacy)	0	5	5	0	1	1	2	0	1	0	0
FGD 5 (medicine)	5	3	5	3	5	0	2	0	0	0	1

* *AUSMAT, Australian Matriculation; FIS, Foundation in Science; UEC, Unified Examination Certificate; STPM, Sijil Tinggi Persekolahan Malaysia*.

From the five focus group discussions, in-depth perspectives on self-regulated learning practice of our students were obtained. By qualitative data analysis, three major themes and seven sub-themes were identified as depicted in Table 2. The major themes generated were “Goal setting as the initiation of SRL practice,” “Adoption of study techniques to assist SRL” and “The role of reflection in SRL.”

**Table 2:** Themes on self-regulated learning.

**Themes**	**Sub-themes**
1. Goal setting as the initiation of SRL practice (*Goals set by the students at the beginning of their study*)	*1.1 Long-term goals* *(Goals to reach at the end of the program or after graduation)*
	*1.2 Short-term goals (Goals to achieve or gain during the program)*
2. Adoption of study techniques to assist SRL (*Different techniques adopted by the students to aid their learning*)	*2.1 Organizing information to facilitate learning (Organization skills)*
	*2.2 Managing self for regulating learning (Management of time, habits etc.)*
	*2.3 Creating a conducive learning environment (Modification of their surroundings)*
3. The role of reflection in SRL (*Self-reflection by the students at the end of each SRL cycle*)	*3.1 Self-evaluation of performance (Self-reflection and analysis)*
	*3.2 Peer guidance (Guidance offered by other students)*

### Theme 1: Goal Setting as the Initiation of SRL Practice

It was evident that across the different programs, the students had entered the university with diverse goals ranging from their ultimate study or work goals to personal improvement goals. Forethought and goal setting is the first step in the initiation of SRL practice that decided their future course of action. Almost all the participants had some goal in mind and had different perspectives on what they wanted to achieve in the course of their university life. This also in turn determined how much time they were willing to spend for academics vs. personal development or extracurricular activities. Personalized goal setting has been known to improve student engagement, learning and academic performance (28).

### Long-Term Goals

Long-term goals are the goals that students want to reach at the end of the program or after graduation. The participants either wanted to work or go for higher studies up to their doctorate degree; some even wanted to become entrepreneurs after completing their degree.

“*I want to work as an employee first and then I build up my experience… and may be (in) future open up business.” (a second-year dietetics student)*.

“*My aim is to …. pursue to my masters which is a more specific course like microbiology; become a microbiologist may be and then get a doctorate degree.” (a second-year biomedical science student)*.

“*I am very interested in doing (sic) a start-up company that is related to medical biotechnology” (a final-year biotechnology student)*.

Most students were focused on their self-development in terms of developing interpersonal skills such as communication, leadership, problem-solving skills etc. to be achieved by the end of their program.

“*My goal is to improve my own communication skills and also my leadership skill … to like problem solving skill I wish I can think independently.” (a second-year pharmacy student)*.

“*I wanted to have more experience in leadership activities and participate um in many extra co-curricular activities*” *(a second-year pharmacy student)*.

The students themselves have realized the importance of qualities or traits that they need to acquire to meet the requirements of the world and have a competitive edge. Hard skills or technical knowledge can only contribute to about 15% of an individual's success, the remaining major chunk of 85% being attributed to soft skills. Of these soft skills, communication skills, problem-solving skills and leadership skills are rated by more than 80% of the students of higher learning institutions to be important (29).

### Short-Term Goals

Their short-term goals are related to the diverse things they wanted to achieve or gain during the program. The goals were either academic goals or social networking or personal goals. Setting academic goals is shown to strongly impact achievement (30). Some students had academic goals related to their performance in the course aiming to achieve a target CGPA.

“*I decided to try and push myself to get above 3.2 CGPA” (a final-year biotechnology student)*.

“*Every semester I will set a target for myself, and I will try to achieve like a CGPA 3.5 or above*.” *(a second-year pharmacy student)*.

Many students were intent on building connections and creating a wide social network during their university life, which they thought would be useful in future.

“*My goal to enter this university (is) to try to meet more people from different background and also uh know people from different cohort(s).” (a second-year pharmacy student)*.

“*When I come to university, I said I'll make more friends … make connections basically know more people” (a final-year biotechnology student)*.

Some of them wanted to have a clear balance between studies and extracurricular activities, wanting both without compromising either.

“*One of the things I really concentrated along is not to let my studies um take over but also not neglect my studies, so making sure that I am actively finding the balance between everything I wanted to do. I didn't want to sacrifice one thing um for another.” (a first-year medical student)*.

“*I think you should really really (sic) enjoy university life and make more friends and gain more knowledge at the same time” (a second-year dietetics student)*.

### Theme 2: Adoption of Study Techniques to Assist SRL

As part of their performance, the students adopted different study techniques to achieve their academic goals, and this added a new element to their self-regulation and approach to study. Another aspect that emerged during the study is that since the students were all undergoing online learning due to COVID-19 pandemic, they were on their own to learn from home and this was an unforeseen disadvantage. They had to monitor their own learning and follow the timetable on their own accord. Many students admitted to not following the university timetable and had to catch up later. This also affected their performance to some extent. In the beginning of COVID-19 lockdown, online learning was perceived to be hard, but the students adapted to the situation over time.

“*During the beginning of MCO, we had to study from home right, and then like I find it difficult to focus” (a second-year biomedical science student)*.

“*To do online learning at first I didn't like it because even to ask a question from a lecturer you have to mail them separately or message them via WhatsApp but now I have I have like got adopted to this new way of learning” (a second-year dietetics student)*.

The participants admitted to missing the university life and socializing with their friends. Some resorted to connecting with their friends through online social media to get some form of interaction.

### Organizing Information to Facilitate Learning

Some learners practiced organization of the information in different ways to suit their personal learning style for ease of learning. The methods were highly personalized, and each had his/her unique method such as creating their own mind maps for each topic, preparing their own study notes, using practice questions for revision and self-check.

“*I try to draw … graphics with like um lines that go down like a family tree and … I use to arrange... different things... and then I go down like a family tree and the lines what are the types... and then what the names and then I have small points as to how they work stuff like that” (a first-year medical student)*.

“*I have my lecture notes, I have some other notes … because you make mind maps, right? So, I do my own notes as well. Because I am using my ipad so I can put in photos I do my own notes” (a second-year biomedical science student)*.

“*I'll do mind maps for each and every chapter, so it is easy for me to go through during the examination” (a second-year biomedical science student)*.

While most participants liked to study alone, some liked to undertake collaborative learning with a group of friends and even tried to maintain it online through social media.

“*I don't really like study in groups … prefer study alone mainly because I'm shy.” (a first-year medical student)*.

“*I do group study because... when I have some doubts, I have to clear them and sometimes what I skip uh I think they (my friends) note that (down) also and... we ask lots and lots of difficult question(s) to him (friends) to clarify that thing (doubts) because uhh that I think those things (clarifying doubts) helps (us) to understand*.” *(a second-year biomedical science student)*.

### Managing Self for Regulating Learning

It also emerged that many students had realized the importance of organizing their tasks and arranging time for different chores to make time for their learning. Some were better than the others in this.

“*I think time management is really important so for what I did was I get a planner … a weekly planner so I write down what I have to do tomorrow, the day after; I will plan out the next 2 days' active task or to do including my personal stuff like going to the gym or cook something*.” *(a second-year dietetics student)*.

“*I have a digital monthly planner so I can see all the deadlines and I can allocate time wisely” (a second-year dietetics student)*.

“*The only problem … is my time management skill. So, sometimes … I procrastinate” (a final-year biotechnology student)*.

They learnt to prioritize their tasks that needed immediate attention or the subjects they needed to study first. Time management was one skill that helped the students manage themselves well and have sufficient free time to pursue their hobbies or relax. Some students were systematic and used calendars and reminders to help them, were consistent in their habits and made a routine.

“*I am making a more effort to actually spread out my time uh and play games and do all these things because I find that when I am not so stressed when I have a method in the house then studying is much easier and a lot more enjoyable” (a first-year medical student)*.

In contrast, there were some students who admitted to having no strategy, did not find studying enjoyable, were lazy and only resorted to studying at the last minute just before the exams.

“*I don't have a study strategy to be honest because I admit that … I am a very lazy person; I really study like last 1 month before exam … when the time is more near near (sic) to the exam then I can feel the pressure then when I have the pressure then my study my effectiveness of study will improve... like I can spend I can spend like 10 h more a day to study that and then I can memorize everything … because I can feel the pressure*.” *(a second-year dietetics student)*.

Volition is a crucial element that affects the students' ability to follow through and achieve the set goals. Procrastination and other dysregulated behaviors are known to affect performance leading to under-achievement (31).

### Creating a Conducive Learning Environment

A conducive learning environment with minimal or non-disruptive noise was considered important for many students to facilitate learning either in the university or their own room. Physical or social environment are part of social determinants of learning and have major influence on higher education (32).

“*A quiet study area that is conducive to study like um library” (a first-year medical student)*.

“*Usually, I will design my own study place even in hostel, I also will design my … table, the chair the wallpaper and all that. I like to design my own study room” (a second-year pharmacy student)*.

All the students were studying from home, and this affected different students differently. Some found the home environment to be more distracting.

“*I get distracted because every time I was like studying, I look out of the window look at the cars, trains and then come back again. So, it takes a lot more time.” (a second-year biomedical science student)*.

“*For me, home is the place that very comfort (able); I can sleep, eat, everything but not study” (a second-year dietetics student)*.

There are a lot of factors that can interrupt the students between their planning phase and actual action. The students admitted to being distracted by their friends, the social media, their smartphones, the apps in the phone, games etc.

“*The phone I think that's the most distracting part when I study, like you know, if you get the notification you feel like checking what it is*.” *(a first-year medical student)*.

“*For me my distraction are those social media the apps because like I will keep scrolling” (a second-year pharmacy student)*.

“*I am easily distracted by my phone, YouTube or Facebook, most of the social media because I will addict to it (sic) and then keep watching them for few hours” (a second-year dietetics student)*.

The successful self-regulators are those who have learnt to deal with distractions and keep them under control. Many students mentioned that they keep the phone away from them while studying or switch on the airplane mode or turn off notifications or keep the phone in silent mode. These strategies prevent them from taking their phone in hand every few minutes. Some even mentioned deleting all the apps and games before semester exams.

“*I restrict myself to not downloading any game to my phone … now so I am only play games or play anything else during my semester break” (a second-year dietetics student)*.

“*When I am studying, I try to place my phone as far away from me” (a second-year pharmacy student)*.

“*I put (my phone) on silent don't disturb … and sit down to work; … my laptop also has do not disturb option, most of the time that works” (a first-year medical student)*.

### Theme 3: The Role of Reflection in SRL

The students engaged in self-reflection after the completion of each task to analyze themselves and review their own performance. The participants seemed to be self-aware, having understood themselves, their ultimate goals, what they want, their strengths and weaknesses and had come to terms with the same. Their course of action was formed after careful consideration of the factors listed above.

### Self-Evaluation of Performance

The students were able to reflect on their performance and identify the reasons for their successes and failures. Self-reflection helps the students to express their feelings, identify their problems thereby leading to better self-understanding (33).

“*Procrastination … I participate in a lot of uh extracurricular activities so um I have a lot of responsibilities la so like after I am done doing all the tasks I need to do I find that I don't really have much time to study.” (a second-year dietetics student)*.

“*Even though I have so many plans its its (sic) not just that I don't plan; I do plan, but actions are not enough, so I always think that I can do more*.” *(a second-year dietetics student)*.

“*After each semester, I will do my self-reflection like figure out what I did well in last semester and so what area that I should improve” (a second-year pharmacy student)*.

Students admitted to procrastination and not studying due to lack of self-discipline or motivation. Some also mentioned missing the interaction with the lecturers or getting a physical book from the library for studying. Some also experienced poor internet connectivity and technical problems with software.

“*I found out I (am) very demotivated then I can't adherent to the timetable… during the MCO (movement control order) because my thought would become because my I would think that ok there's no need for me to attend that specific time I can study whenever. Ok so these will even like increase my my procrastination because … I prefer face to face because like it really will push me ok I have to go ya*” *(a second-year dietetics student)*.

Some students on the other hand, liked online learning better than face to face since it allowed them greater control of their learning. Many students were comfortable with recorded lectures since they could pause and rewind multiple times. It also saved travel time for students who lived far from the university.

“*Now I actually prefer like online lectures yea because uh we can record, and we can revise back and actually we can hear it clearly” (a second-year pharmacy student)*.

### Peer Guidance

The students also attached a lot of value to guidance from their friends who pointed out their errors or correct or guide them. Friends also served as major motivating factors in keeping them on the academic track, guiding them on study techniques and chiding them when they played truant.

“*My friends did teach me effective study methods back when I was so struggling how how (sic) to figure out how to study*.” *(a final-year biotechnology student)*.

“*What motivates me to study is my friends whenever I see my friends start studying then I will start to study” (a final-year biotechnology student)*.

Interestingly, the participants said they were receptive to the suggestions and guidance offered by their peers and accepted their perspectives for self-improvement. During the lockdown, they mentioned the lack of interaction and motivation from friends. Some admitted to connecting online and studying at the same time to give a semblance of togetherness.

The students had gone through the full cycle of SRL involving forethought, task analysis and goal setting before joining the university. As they were progressing through their studies, they used various strategies and study techniques to achieve their preset goals. At the end of each semester, they were found to engage in self-reflection and analysis. Our study gives an insight into our students' thought processes, activities as well as the challenges they face during the course of their study.

## Discussion

Our study indicates that students from different undergraduate programs are practicing self-regulation in different aspects of their journey through the university in terms of goal setting, deciding on strategies to assist SRL and self-reflection. By deciding beforehand what they want to achieve, they could follow through in their own way. Our students' self-regulation fits into the cyclical phases model of self-regulated learning proposed by Zimmerman which are- forethought, performance and self-reflection (1). Forethought phase encompasses preparation for the task and task analysis which our students have done before entering the university. Performance phase includes carrying out the task with self-control as well as self-observation. Self-reflection phase involves self-judgement and self-reaction leading to evaluation of one's own performance. Our students appear to follow this cycle of SRL, with goal setting, performance, self-monitoring, and self-reflection. All the phases of SRL are sufficiently reflected in the identified themes. At the end of each task, in our case each semester, their findings influence the forethought in the next cycle. Students from all the programs showed evidence of SRL practice. Our findings are similar to SRL practiced by medical students in the clinical context where diversity in the use of SRL strategies was found to be linked to individual goals set as well as by contextual and peer factors (20). Our study has confirmed the interaction between individuals and the context in determining SRL practice.

According to the attribution theory on cause and effect (34), the students' study behavior is influenced by their future plans as well as their own successes or failures. They fix their performance goals based on what they want to achieve in academics (35). Performance goals or mastery goals were set by highly motivated students who set their bars high and aimed at getting high marks as well as improving their knowledge and skills (36). Some of our students had personal development goals and were inclined toward earning their degree with minimal effort and time. It has been documented that complete dependency on test scores no longer would vouch for future jobs of current learners but rather creativity and non-cognitive skills would be playing a critical role in determining their future success (37). Current global job market seems to place more demand on soft skills such as critical thinking, communication and problem-solving skills (38), the very skills our students have set as their goals in achieving through university education.

Building social networks is a necessity in today's world leading to more job and business opportunities (39). These networks connecting diverse people are known to be crucial in career growth, advancement as well as accomplishments. Apart from personal networks, professional networks like LinkedIn are known to directly help build an online portfolio for job search as well as career advancement. Our students seem to have realized the importance of having their own extended network and therefore were keen on starting to build their networks during their university life. They do not have social inhibitions and are all set to harvest the benefits of networking (40). This emphasizes the importance of career development units in universities which provide students with job opportunities as well as access to networking with professionals and industry partners.

Study-life balance is recognized as another major factor affecting academic performance as well as mental health (41, 42). Students are advised against promoting academics at the cost of personal factors such as relationships, sports, culture, and social life. Time management strategies can help the students achieve this balance (43). Many of our students spoke about choosing to have a balance in life between their academic and other pursuits as demonstrated in setting of short-term goals. This shows their mental maturity, their ability to manage their life with an awareness that would help reduce stress and revitalize them. The concept of study-life balance is addressed in universities of late and support is given to students in terms of job opportunities as well as flexible hours. Given that students might not seek support or be aware of the available facilities, it's important that student services departments in universities highlight and promote these facilities to the students.

Our study provides a new perspective of SRL practice by university students during COVID-19 pandemic. The students were learning from home, away from faculty and friends, deprived of the study atmosphere, which posed more challenges to their practice of SRL. This made the students realize that they had to practice some form of self-regulation if they were to succeed in their academics to overcome the increased stress, distractions at home as well as limited social support (44). This was evident in our study where the students indicated they did not have the conducive quiet learning environment or support from friends, which affected their performance. Online distance learning, while giving the students greater flexibility in scheduling their learning activities, also places the onus on the students to self-regulate their learning and stay motivated.

Self-control is considered a crucial factor contributing toward subjective academic achievement much more than the cognitive ability of the students (45). This finding is corroborated by our study and elaborated under “adoption of study techniques.” Our students have discussed the different techniques they adopt to keep themselves focused on achieving their goals. Self-control is the voluntary regulation of impulses and has a direct correlation to students' success in future (46). Students leaning toward non-academic pursuits, while aiming to pass the exam, started their preparation very late just to ensure that they passed the examinations. Studies have pointed out differences in the practice of SRL between high achievers and low achievers, which was also noted in our study (16). Different strategies are used by the students to stay focused and achieve their respective goals. The use of SRL strategies by the students are diverse and linked to their goals, contextual factors as well as social factors (20), as observed in our study. These studies present learning as a social and interpersonal process that is influenced by multiple social, emotional, and interpersonal factors along with cognitive factors. Knowledge, cognitive strategies as well as self-management skills are indicated to be important factors indicating college readiness in students (47). Time management skills are positively correlated to academic achievement and lower anxiety levels; this coupled with effort regulation and critical thinking was linked to academic success (48–50). In the absence of self-regulation, passive procrastination was evident in some students leading to negative consequences in terms of academic performance. Especially during online learning, those with good time management strategies had better performance than the rest (51). Environment structuring is another factor that has long been known to play a crucial role in promoting or inhibiting SRL. Selecting and modifying the learning environment and adopting different study strategies are behavioral processes that are evident in proactive learners (52). Learning efficiency is established to peak in ambient environments with good lighting and quietness (53). Noise has been perceived as one of the major factors affecting cognitive performance in studies (54) and is given priority by our students in deciding their preferred study place.

Certain negative influencers of SRL also emerged in our study. Either the physical environment or the friends or the social media, this generation of learners have a lot to deal with before nearing their goals. Studies have shown a negative correlation between academic performance and time spent on online social media. A study among university students showed more than 50% of them were addicted to social media and this affected their academic performance (55). During the COVID-19 lockdown and the subsequent prolific use of digital media, the students found themselves spending more time than usual on online social media and games. Those who spent most of their time online for such activities lagged behind academically and had to go for last-minute cramming for exams adopting a surface-learning approach (56, 57). Addiction to smart phones and social networking has also been linked to a variety of psychological problems. The students in our study have admitted to spending more time on social media to the point it was interfering with their academic performance. Mobile phone use and multitasking with the phone are known to disrupt rather than promote academic performance. Learners are distracted by the ring of the phone, texting as well as social media (58). This has been acknowledged by our participants who adopt various strategies to avoid this distraction. When students deploy strategies at an early stage toward modifying their situation to stop themselves from succumbing to a momentarily rewarding impulse are more successful (59). Fostering SRL among the students can make a difference in their life and this initiative is currently being adopted by universities.

As the students had to undertake more online learning due to COVID-19, they had to adapt to the autonomous learning environment by devising strategies to discipline themselves to follow the timetable and learn independently. Initially, the students faced some difficulties with the sudden move into online learning but they learnt to overcome them by following time management and effective use of online resources to support their learning (60, 61). Distance learning required that students autonomously learn to practice SRL and devise their own support systems (18). One study classified students into different categories based on their resource management. Most students were “maintainers” who maintained their performance level by regulating their time and effort. Some were “overwhelmed” or “surrenderers” who had difficulty adapting to online learning. The “adapters” were the most adaptive to the situation whose performance increased owing to their increased efforts (62). Our students, once they adapted to online learning, welcomed having online lectures as a permanent feature in feature since it allows them the flexibility of scheduling their study, individualize the learning process and revisit the content at their own time and pace. Between synchronous and asynchronous learning, while the former provides more personal interaction, the latter allows the students flexibility in terms of time as well as other factors like internet connectivity. They could learn anywhere, anytime with asynchronous mode and this suited them if they had to work or care for their family (63). As academics, we can help them by creating flexible timetables, offering both synchronous and asynchronous learning methods, digitalizing learning resources and providing collaborative learning environment to give greater flexibility to students who might opt for on-campus or off-campus learning thereby facilitating active student engagement.

Another irrevocable factor that emerged with online learning is that the students missed social interactions with their peers as well as lecturers. While this feeling is common during the lockdown, the same has been reported earlier with online blended learning (64). Moving forward, increasing collaborative learning online as well as providing a comfortable interactive atmosphere where the students feel connected are essential to ensure the mental wellbeing of our students. Conducting synchronous sessions with live chat would also maintain some level of contact with the teachers and peers. Our students had gotten used to contacting their teachers as well as friends online and adapted well to the situation.

Our study has shown that SRL is practiced widely by undergraduate students in our university. Although individual students might set different goals with different outcome expectations, they show evidence of practicing SRL to get the best out of their university life. Our study gives an insight into SRL practice, the expectations of the students as well as the challenges faced by them and adds to the body of literature on SRL especially in the context of online remote learning. Moving forward, in future, we can promote SRL practice among the students, provide guidance and support especially in the early semesters to help them be independent learners. Faculty can also adapt to managing the expectations of the students to facilitate academic tutelage as well as personal development, both in online and face to face learning environments. Future research could focus on studying the impact of online and blended learning on SRL practice of the students and the changes in SRL practice that aid our students in adapting to remote learning. It can involve students from multiple universities and focus on how students' perception and behavior changes over time.

### Limitations

This is a qualitative study that gives only a snapshot of the perspective of the students at that time. This study is not intended to compare the students from different programs but rather is aimed at getting their perspectives and practice of SRL. This study was conducted in only one university in Malaysia. Because of the pandemic, the students' learning experiences was limited to online learning and their self-regulation might have changed. The changes could have been explored in depth.

### Implications

This gives a view of student behavior and SRL practice in undergraduate settings that can be applied to any university. This study identifies the important factors to consider in offering the current students a satisfying university learning experience.

## Conclusion

The key findings of the study are that undergraduate health professions students initiate SRL practice by setting their own academic as well as personal goals when they come to the university. They adopt various strategies in context to their settings to regulate their learning followed by self-reflection and retrospection. Self-regulation was a crucial factor that determined their success especially in the context of online learning environment imposed by COVID-19 induced lockdown. The students were able to adapt and modify their tactics to achieve their goals and practice SRL. This study gives an insight into the various expectations of the students in terms of personal and professional development and on online learning behavior. Our study suggests that by promoting an atmosphere of SRL in universities we can help them achieve their goals and be successful in their careers.

## Data Availability Statement

The raw data supporting the conclusions of this article will be made available by the authors, without undue reservation.

## Ethics Statement

The studies involving human participants were reviewed and approved by IMU Joint Committee on Research and Ethics. The patients/participants provided their written informed consent to participate in this study.

## Author Contributions

All authors listed have made a substantial, direct, and intellectual contribution to the work and approved it for publication.

## Funding

This study was supported by the International Medical University under the Grant MHPE I/2020(04).

## Conflict of Interest

The authors declare that the research was conducted in the absence of any commercial or financial relationships that could be construed as a potential conflict of interest.

## Publisher's Note

All claims expressed in this article are solely those of the authors and do not necessarily represent those of their affiliated organizations, or those of the publisher, the editors and the reviewers. Any product that may be evaluated in this article, or claim that may be made by its manufacturer, is not guaranteed or endorsed by the publisher.
